# Alcaligenes faecalis, Stenotrophomonas maltophilia, and Pseudomonas aeruginosa in a Mixed Microbial Contact Lens Keratitis: A Case Report

**DOI:** 10.7759/cureus.76202

**Published:** 2024-12-22

**Authors:** Shah Nadeem Ahmad, Abhinav Loomba, Sid Goel

**Affiliations:** 1 Ophthalmology, St James’s University Hospital, Leeds, GBR; 2 Ophthalmology, Hull University Teaching Hospitals NHS Trust, Hull, GBR

**Keywords:** alcaligenes, contact lens, cornea, keratitis, stentophomonas

## Abstract

We present a case of mixed microbial keratitis in an otherwise healthy contact lens wearer. The microbes detected on microscopy included *Pseudomonas aeruginosa*, *Acanthamoeba*, *Alcaligenes faecalis*, and *Stenotrophomonas maltophilia*. *P. aeruginosa* and *Acanthamoeba* are well-recognised corneal pathogens, although *S. maltophilia* is uncommon, and *A. faecalis* is extremely rare. We were only able to find one previously reported case of *A. faecalis* keratitis, in a cornea that was otherwise compromised by ocular cicatricial pemphigoid. To the best of our knowledge, this case represents the second reported case of *A. faecalis* keratitis, and the first one in the context of a mixed microbial contact lens-related keratitis.

## Introduction

Contact lens keratitis is a common, potentially sight-threatening presentation to emergency eye services and can be caused by a variety of pathogens, including bacteria, viruses, fungi, and amoeba [1]. Accurate identification of the causative pathogens and their antimicrobial sensitivity is important for directing treatment in severe cases. Here, we describe a case of mixed microbial contact lens keratitis, with three causative organisms (*Pseudomonas aeruginosa*, *Stenotrophomonas maltophilia*, and *Alcaligenes faecalis*), one of which (*A. faecalis*) is an extremely rare cause of keratitis and required oral antimicrobial treatment rather than topical treatment.

## Case presentation

A 40-year-old female patient presented to the emergency eye service with a one-day history of right eye pain, redness, and foreign body sensation. The day before, she had gotten some glitter in her eye, which was removed at home with a cotton bud, and had later inserted her normal contact lenses. The patient had good contact lens hygiene. The current set of contact lenses was three weeks old.

**Figure 1:**
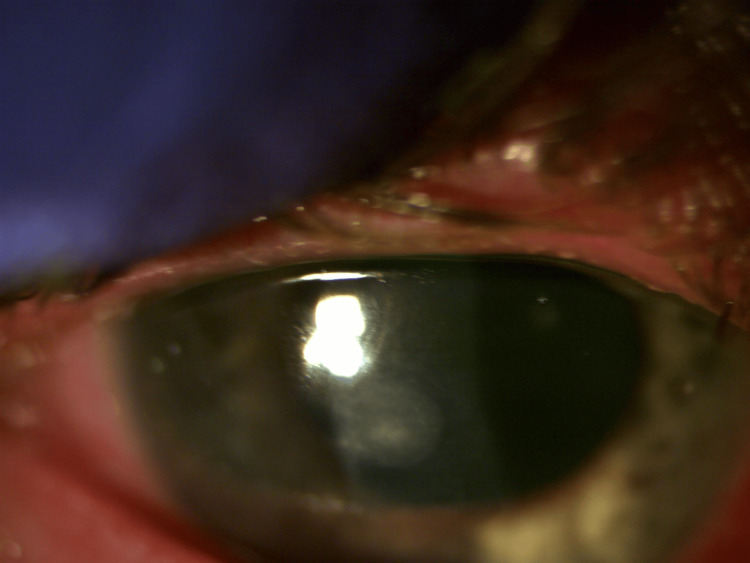
Appearance of the cornea on day 4, showing a paracentral corneal ulcer and conjunctival injection.

On initial presentation, she was noted to have a corneal infiltrate measuring 0.9 x 0.7 mm with spoke-like extensions. She was started on hourly topical ofloxacin 0.3% drops to her right eye, to be instilled day and night, and returned for review the next day. On day 2, her vision had dropped to hand movements, with no improvement with pinhole, and she had a 0.7 mm epithelial defect and infiltrate, as well as a 0.7 mm hypopyon with clear vitreous and no evidence of endophthalmitis. A corneal scrape was sent, and her contact lenses, contact lens case, and contact lens solution were also sent for microbiology. Her treatment was switched to preservative-free levofloxacin 0.5% and preservative-free cefuroxime 5%, both to be used hourly, day and night, and oral doxycycline 100 mg daily. Initial corneal appearances on day 4 can be seen in Figure 1.

Microbiology investigations obtained from the contact lenses and casing, returned on day 9, revealed a heavy growth of *Pseudomonas*, sensitive to ceftazidime and gentamicin; *A. faecalis*; *S. maltophilia*, sensitive to trimethoprim-sulfamethoxazole; and negative fungal and viral cultures. On obtaining these results, her treatment was switched to hourly cefuroxime 5% and gentamicin 1.5%, and oral trimethoprim-sulfamethoxazole was added. By day 12, the visual acuity had improved to 6/18 (6/12 with pinhole). The patient's epithelial defect had resolved, and the infiltrate had been replaced by scarring. At this point, the topical antibiotics were each reduced to six times a day, and topical prednisolone 1% TDS was added.

**Figure 2:**
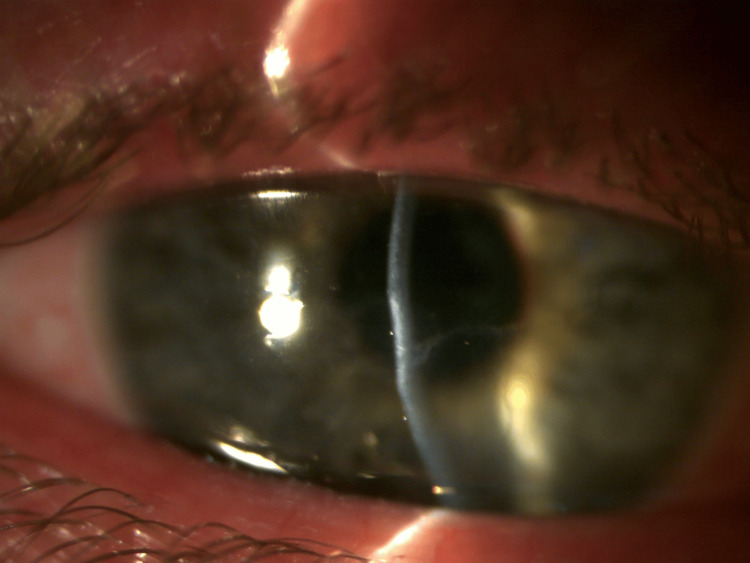
Cornea after three months. There is a faint central circular and linear scar with some associated stromal thinning.

The result from the *Acanthamoeba* specimen was delayed and came back positive on day 22. At this point, the patient was clinically stable, and there were no overt signs of *Acanthamoeba* infection. Although acting on the side of caution, the steroids were stopped, polyhexamethylene biguanide (PHMB) and Brolene were started two-hourly, and the cefuroxime and gentamicin were reduced to QDS. Following continued improvement, on day 43, the PHMB and Brolene were reduced to three-hourly, and prednisolone 0.5% preservative-free was added BD. Treatment was tapered down until day 81, when topical antimicrobials were stopped. The final visual acuity was 6/18 (6/12 with pinhole), due to a central corneal scar. The corneal appearance on the resolution of the infection is visible in Figure 2.

## Discussion

Our patient had a mixed microbial keratitis, comprising *P. aeruginosa*, *A. faecalis*, *S. maltophilia*, and *Acanthamoeba*, although it must be noted that all these results were from the contact lens and the contact lens fluid, rather than from the corneal ulcer itself (the initial corneal scrape on day 2 was done while the patient was already taking hourly ofloxacin 0.3% eye drops). Her corneal ulcer developed after epithelial trauma from a foreign body and its subsequent removal at home with a cotton bud, shortly after which the patient re-instilled her contact lens. Epithelial trauma is a known risk factor for the development of a contact lens ulcer [1].

*P. aeruginosa* is a well-known gram-negative bacterium and is the most common bacterium implicated in contact lens keratitis [2]. *S. maltophilia* and *A. faecalis* are less common causes of corneal ulcers.

*S. maltophilia* is a gram-negative organism that has a propensity to form biofilms and survive in nutrient-poor environments. It is mainly implicated in lower respiratory tract and bloodstream infections [3]. It is a rare cause of keratitis, and when it is isolated, it usually occurs as an opportunistic pathogen in corneas that have an otherwise compromised ocular surface, either due to trauma, contact lens wear, or infection from another pathogen, including viral pathogens [4-6]. Previously published case reports and series report sensitivities to fluoroquinolones [5], which have also been shown to be effective in respiratory tract and bloodstream infections when given systemically, although our case had sensitivities to trimethoprim-sulfamethoxazole, which is considered first-line antimicrobial therapy for *S. maltophilia* infections elsewhere in the body [7]. Our case was initially treated with topical fluoroquinolone and was later started on oral trimethoprim-sulfamethoxazole once antimicrobial sensitivities were detected.

*A. faecalis* is an aerobic gram-negative bacillus and is an extremely uncommon cause of ocular infection. Systemically, it has been known to affect the bloodstream, urinary tract, skin and soft tissue, and middle ear. It is multi-drug resistant but is most commonly sensitive to imipenem, meropenem, and ceftazidime [7], as well as high-dose tigecycline [8]. When it is detected, it is often one pathogen in a mixed microbial infection [7].

There is one previously reported case of *A. faecalis* keratitis in 1993 [9], where the patient's corneal scrapes were initially positive for *Serratia marcescens* and *Staphylococcus aureus*, but, due to poor response to treatment, a further corneal scrape on day 12 showed *A. faecalis*. In this case, the ocular surface was compromised by cicatricial pemphigoid. Importantly, this patient was admitted to the hospital for inpatient treatment; *A. faecalis* is commonly reported as a hospital-acquired infection [8,9]. This case differs from ours in several details: our patient did not have any ocular comorbidities, whereas the previously reported case had longstanding ocular cicatricial pemphigoid. Additionally, our patient contracted *A. faecalis* in the community, rather than in the hospital. The comorbidity of cicatricial pemphigoid is one main factor in the poor outcome of this previously reported case, which resulted in significant corneal stromal loss and required cyanoacrylate glueing. Our patient's condition did not reach this level of severity.

One case of endophthalmitis due to *A. faecalis* has been reported in 2002, which occurred four days after a penetrating keratoplasty. Here, *A. faecalis* was found in isolation and was sensitive to polymyxin B, amikacin, tetracycline, and partly sensitive to ciprofloxacin [10].

A related pathogen, *Alcaligenes xylosoxidans*, has been known to rarely cause corneal infections [11,12]. *A. xylosoxidans* shares many characteristics with *A. faecalis - *both bacteria are aerobic gram-negative bacilli, and both are associated with soil or water. Published cases of *A. xylosoxidans* keratitis occur in already-compromised corneal surfaces or on ocular surfaces immunocompromised by topical steroids. A variety of antibiotic regimens have been tried. Like *A. faecalis*, it shows multi-drug resistance, commonly resistant to aminoglycosides and cephalosporins, with varying sensitivity to fluoroquinolones.

## Conclusions

Our patient presented with a mixed keratitis consisting of *P. aeruginosa*, *S. maltophilia*, and *A. faecalis*. Of these, *S. maltophilia* and *A. faecalis* are rare causes of ocular infection; *A. faecalis* keratitis has only been reported once before in the literature. *S. maltophilia* and *A. faecalis* often occur on compromised ocular surfaces and may act in an opportunistic way. Our patient was a contact lens wearer and had a foreign body in her eye shortly before instilling her contact lenses - this may have resulted in the compromised corneal surface, allowing a mixed microbial keratitis involving opportunistic pathogens to develop.

Antibiotic sensitivities for *A. faecalis* are variable in the literature, although our case of mixed microbial keratitis improved with the empirical treatment of topical cefuroxime and gentamicin, as well as oral doxycycline and trimethoprim-sulfamethoxazole.
